# The need for data in the world’s most violent country

**DOI:** 10.2471/BLT.14.136713

**Published:** 2014-10-01

**Authors:** Marie-Renée B-Lajoie, Shawn D’Andrea, Cristina Rodriguez, Gregg Greenough, Cristina Rodriguez, Ronak Patel

**Affiliations:** a Family Medicine, McGill University, 5858 Chemin Côté-des-neiges, 3rd floor, Montreal, H3S1Z, Canada.; b Emergency Medicine, Brigham and Women's Hospital, Boston, United States of America (USA).; c Hospital Escuela Universitario, Tegucigalpa, Honduras.; d Harvard Humanitarian Initiative, Boston, USA.

In Honduras, there were 9453 violent deaths reported in 2013, an average of one violent death per hour.^1^ Honduras has been ranked the most violent country on the planet. The data available on homicides in the country are limited but indicate rates as high as 318.3 homicides per 100 000 among men aged 20–24 years in 2013.^1^ San Pedro Sula and Tegucigalpa are considered to be the first and fifth most violent cities in the world, respectively.^2^ The rates of violent death in Honduras surpass those observed in many war-affected countries. Elevated rates of homicide and violence unrelated to warfare are not, however, confined to Honduras but also occur in densely urbanized areas in several other countries, including cities in Brazil, Mexico, Pakistan and South Africa.^2^

Honduras lies in a region known as the northern triangle of Central America, along with El Salvador and Guatemala. Its population, estimated at slightly more than eight million in 2012, relies on a fragile economy. The soaring rates of violence seen over the last decade have been attributed to political instability, inequality, corruption, organized crime and the rise of several gangs or *maras*. The truce that two of the major *maras* signed in May 2013 has yet to have a substantial impact.

Homicides represent only a part of the violence experienced by the Honduran population. Traumatic injuries, sexual violence and the psychological sequelae of violent acts contribute to negative health outcomes. In 2012 the Hospital Escuela Universitario – the main academic trauma referral hospital for the city of Tegucigalpa – received almost 20 000 cases of traumatic injury and these cost about eight million United States dollars to treat.^3^^,^^4^ Approximately 15% of these people had been assaulted, stabbed, shot or otherwise intentionally injured. In the only study investigating homeless individuals from Tegucigalpa, Médecins Sans Frontières found in 2007 that of every 10 street-based persons, two claimed to have suffered severe physical violence – and another four claimed to have suffered milder forms of physical violence – in the year before interview.^5^ Many of the subjects exposed to severe violence had never presented to a health-care facility and many of the homeless individuals investigated had mental health and substance abuse problems.^5^

The data that are available on violence in Honduras tend to be inaccurate and incomplete. Some relevant data come from the country’s medical forensic and police departments but inconsistency in reporting makes extrapolation of these data difficult. A general shortage of data on trauma and its consequences – in Tegucigalpa, elsewhere in Honduras and in violence-affected cities in many other countries – hampers a broader analysis and the design of effective programmes and policies for violence prevention.

The aim of the *Observatorio de la Violencia* – a project led by the rector of the Universidad Nacional Autónoma de Honduras – is to generate comprehensive and reliable data on violence in Honduras. At the time of writing, the best data come from the records of the country’s forensic medicine, police and public transport departments. Although it would be useful if every health-care facility could keep a trauma registry, the Hospital Escuela Universitario is the only facility in Honduras that has routinely collected data on trauma cases during the last decade. Even though violence in Honduras is assumed to be associated with huge social and financial costs, there have been no concerted, sustainable and national attempts to reduce violence and the effects of violence in the country. Frequent staff changes in the national ministry of health, which have resulted in the appointment of eight ministers of health in eight years, have hampered the development of effective and sustainable programmes of violence prevention.

There is an urgent need to respond to the growing levels of violence in Honduras. Appropriate programmatic and policy solutions need to be driven by more consistent, comprehensive and frequent collection of data on the problem. The usefulness and value of the *Observatorio de la Violencia* need to be increased by data aggregation and analysis and by the routine collection of data on violence-related injuries at community level. Once the main risk factors and the most vulnerable populations have been identified, effective interventions based on risk reduction can be focused on the groups that are at greatest risk. A national, multidisciplinary violence-response network needs to be developed. The Honduran health-care system must move beyond stitching and splinting to become a central pillar in the collection of data on violence and a main player in any programme of violence prevention. As the problem of rising urban violence is not confined to Honduras, similar policies to evaluate violence-related mortality and morbidity may need to be implemented in other countries.
